# Sleep regularity, circadian rhythms, and chronotype as mechanisms of risk for affective dysregulation in female adolescents

**DOI:** 10.1017/S0954579426101242

**Published:** 2026-02-25

**Authors:** Christopher Sikes-Keilp, Kayla A. Jensen, Elizabeth D. Wilson, Jessica R. Lunsford-Avery, Elizabeth H. Andersen

**Affiliations:** 1 Psychiatry, The University of North Carolina at Chapel Hill, USA; 2 Psychology and Neuroscience, The University of North Carolina at Chapel Hill, USA; 3 Psychiatry and Behavioral Sciences, Duke University, USA

**Keywords:** Adolescent sleep, chronotype, circadian rhythms, mood, sleep regularity

## Abstract

In a sample of early post-menarchal female adolescents, this study examined sleep regularity in relation to depression symptoms, circadian rhythms, and chronotype preference. Sixty-six female adolescents, aged 11–14 and within fifteen months post-menarche, completed a one-week sleep and circadian rhythm assessment involving self-reported sleep behaviors, 24-h sleep monitoring using wrist actigraphy, and serial cortisol and 6-sulfatoxymelatonin collections for four days. Sleep regularity was operationalized as the probability of being in the same wake/sleep state at any two timepoints 24 h apart (i.e., sleep regularity index (SRI)). Reduced SRI was associated with higher depressive symptoms (*F* [1,273] = 18.65, *p* = < .0001), as were eveningness chronotype (*F* [1,273] = 21.13, *p* = < .0001), sleep duration (*F* [1,273] = 6.25, *p* = .01), and self-reported life stress (*F* [1,273] = 22.82, *p* = < .0001). The interaction between SRI and chronotype was also a predictor of increased depression (*F* [1,273] = 18.65, *p* = < .0001), such that eveningness and low sleep regularity predicted higher scores. Sleep regularity was not significantly associated with cortisol awakening response, cortisol slope, or overnight melatonin levels. Sleep regularity appears linked to altered mood in early post-menarchal girls. Further research linking sleep regularity to physiological processes governing sleep is warranted. Interventions targeting sleep regularity stand to improve mental health outcomes, as well as promote healthy developmental trajectories for affect regulation.

## Introduction

Mood and anxiety disorders affect 15%–25% of adolescents (Racine et al., 2021) and are associated with social impairment, poor academic performance, substance use, obesity, and death by suicide (Bitsko et al., 2018). Sleep disturbances occur in 60%–80% of adolescents with major depressive disorder (MDD) and generalized anxiety disorder (GAD) (Chase & Pincus, 2011; Liu et al., 2007), with experimental evidence suggesting poor sleep may be one causal factor underlying affective dysregulation in this age group (Baum et al., 2014; Booth et al., 2021). It is also well-established that sex differences in rates of mood and anxiety disorders emerge during adolescence (Angold et al., 1999), increasing in relative prevalence to approximately twice the risk for female adolescents. These findings suggest that sleep disturbances during adolescence may contribute to sex differences in mood pathology. While prior studies have established insufficient sleep duration as a predictor of affective dysfunction in mixed sex samples (Johri et al., 2025), research is critically lacking in several areas, including: (1) *a priori* examination of sex-specific effects; (2) consideration of additional sleep and sleep-related characteristics that are directly influenced by puberty, such as sleep regularity and circadian timing; and (3) characterizing outcomes by specific stages of adolescence, particularly the pubertal transition, where vulnerabilities to mood and anxiety disorders emerge.

### The pubertal transition

As children move from prepuberty into puberty, their natural sleep and wake times shift later, reflecting an average delay in circadian timing (Laberge et al., 2001). This delay occurs at an earlier age in female compared with male adolescents, and is associated with altered brain development (Lunsford-Avery et al., 2020) and mood symptoms (Liu et al., 2007). Additionally, several sex differences in sleep function emerge during puberty, with adolescent females experiencing higher rates of primary sleep disorders (Marver & McGlinchey, 2020), shorter sleep duration (Maslowsky & Ozer, 2014), poorer sleep quality (Galland et al., 2017), and higher levels of daytime fatigue (Forest et al., 2022). These differences can be attributed in part to activational (or acute) effects of gonadal steroid hormones – estradiol/progesterone for females, testosterone for males – as these hormones directly modulate sleep processes (Mong & Cusmano, 2016). Further, interactions between gonadal hormones, the central circadian pacemaker, and the hypothalamic-pituitary-adrenal (HPA) axis, which coordinates physiological responses to the psychosocial and other stressors experienced during adolescence, play a major role in sleep and affective function (reviewed in [Bailey & Silver, 2014]). Experimental evidence suggests that this integrated system may be more susceptible to dysregulation in females, particularly in the context of heightened stress (Chen et al., 2006; Verma et al., 2010).

Endogenous (e.g., hormone exposure) and exogenous (e.g., life experience) processes also exert *organizational* effects during puberty (Schulz et al., 2009), shaping physiological systems in a longer-lasting fashion during sensitive developmental windows. Organizational effects during puberty are highest near the pubertal transition (Schulz et al., 2009) and are necessary for healthy maturation of brain networks governing emotion-regulation and cognition. Studies have suggested that adolescent sleep disturbance can not only produce acute deficits in mood, but may also adversely affect developmental trajectories and increase the risk of mental illness in adulthood (Roane & Taylor, 2008; Uccella et al., 2023), consistent with direct organizational effects of sleep disturbance. The mechanisms underlying these effects – for instance, how they interact with/influence normative pubertal organizational effects of gonadal steroids to yield sex differences in pathology – are poorly understood. Nonetheless, understanding the sex-specific influence of sleep and circadian processes during sensitive periods such as puberty may have substantial implications for both *treatment* and *prevention* of mental illness.

### Sleep-related processes and mood

Sleep is facilitated by two parallel processes: (1) a homeostatic drive for sleep and (2) circadian rhythms (Borbély et al., 2016). The former increases with time spent awake; sleep of insufficient duration does not fully reset the homeostatic drive, resulting in excessive daytime sleepiness (Durmer & Dinges, 2005). Circadian rhythms increase and decrease wakefulness within ∼ 24-h oscillations anchored to environmental cues (e.g., light and daily routines) (Rosenwasser & Turek, 2015), and altered circadian rhythms may manifest as wakefulness or arousal at atypical times (e.g., late evening – a circadian *phase delay*, or very early in the morning – a *phase advance*) (Pavlova, 2017).

Sleep deprivation and altered circadian timing have both been associated with mood impairment during adolescence (Johri et al., 2025). Additionally, individual variability in circadian entrainment, known as chronotype, can interact with circadian timing to exacerbate phase disturbances and associated behavioral outcomes (Zou et al., 2022). Chronotype can generally be categorized into *morningness* (a tendency toward greater arousal during the morning) and *eveningness* (a tendency toward greater arousal during the evening). Substantial evidence (rev. in [Kivelä et al., 2018]) has linked eveningness chronotype with psychopathology (e.g., depression, anxiety, psychosis, and maladaptive eating behaviors). Multiple biological systems have been implicated in affective dysfunction related to circadian phase misalignment, including changes in hypothalamic-pituitary-adrenal (HPA) axis (Nicolaides et al., 2000), inflammatory (Zielinski & Gibbons, 2022), and melatoninergic (Honma et al., 2020) function.

### Sleep regularity and adolescent mental health

Another emerging feature of sleep that appears important for affective function is *sleep regularity*, defined by the sleep regularity index (SRI) as the probability of an individual being in the same wake/sleep state at any two time points 24 h apart (Fischer et al., 2021). Irregular sleep patterns are common in adolescence (e.g., decreasing weekday sleep to meet academic demands, compensating by increasing weekend sleep), and reduced sleep regularity during adolescence has been associated with psychiatric symptoms (Bei et al., 2017; Castiglione-Fontanellaz et al., 2023), including depression (Lunsford-Avery et al., 2022). Sleep regularity has been shown to improve prediction of adolescent behavioral health outcomes beyond shortened/fragmented sleep (Lunsford-Avery et al., 2022), and inconsistent associations with other sleep measures suggests that sleep regularity is regulated by unique physiological processes (which may bear specific interactions with puberty). Though not utilizing SRI specifically, one study found that variability in sleep minutes predicted trajectories of internalizing and externalizing symptoms in a longitudinal study of children and adolescents, consistent with the idea that sleep regularity may have long-term effects on affective development (Thompson et al., 2024). It should be noted that some research suggests that irregular sleep across the lifespan may be related to desynchrony of circadian rhythms (Murray et al., 2019; Phillips et al., 2017).

Given the apparent influence of sleep on short- and long-term behavioral outcomes, sleep regularity during puberty may serve as an important metric for predicting both the development of acute mood symptoms as well as risk of adulthood psychiatric disease. However, no studies to date have examined sleep regularity in relation to mood during puberty specifically. Further, despite strong evidence that sleep modulates biophysiological function (Morris et al., 2012), little work has directly examined how sleep regularity influences the circadian release of hormones such as cortisol and melatonin. Lastly, relationships between sleep regularity, biological sex, and mood have not been established.

### The current study

The present study assesses relationships between sleep regularity, mood, and circadian rhythms using actigraphy-based sleep data obtained from a larger study examining hormone-mood relationships in early post-menarchal (within 15 months of menarche) female adolescents. The primary aims of this sub-study were to examine: (1) sleep regularity as a predictor of depressive symptoms; (2) the impact of chronotype on the relationship between mood and sleep regularity; and (3) sleep regularity in relation to hormone measures of circadian function, including diurnal cortisol and 6-sulphatoxymelatonin (aMT6s), a urinary metabolite of melatonin. We hypothesized that sleep regularity would predict average depression scores across the study period, and that chronotype would moderate the effects of sleep regularity and mood given the generally protective effect of morningness against affect dysregulation (Zou et al., 2022) and findings suggesting that chronotype may moderate sleep effects on behavior/cognition (Wang et al., 2024). For circadian hormones, we hypothesized that reduced sleep regularity would be associated with a flattened diurnal cortisol curve, reduced cortisol awakening response, and decreased morning aMT6s levels (which reflect melatonin from the previous evening).

## Methods

### Participants

Adolescents (*n* = 66) aged 11–14 who were assigned female sex at birth and undergoing a healthy pubertal transition were recruited using flyers, mass emails to community members, and electronic school communications. Participants were mid-to-late puberty (Tanner Stage 4) according to self-assessment via line drawings and within 15 months post-menarche, as indicated initially by caregivers during screening, and confirmed by self-report on the *Pubertal Development Scale* (PDS). Psychotic symptoms, bipolar disorder, or active suicidal ideation were exclusionary, as were medications or supplements known to alter hormones, mood, and neurological function (e.g., hormonal contraceptives, antidepressants, anxiolytics, and stimulants).

### Procedure

**Figure 1.**
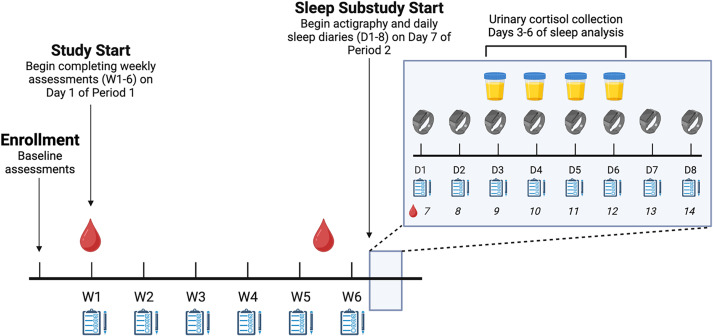
Overview of Study Design. Participation began on Day 1 of participants’ first menstrual period following enrollment (Period 1) and continued for a total of 6 weeks. Starting on Day 1 of Period 1, participants completed 6 weekly surveys assessing mood, stress, and sleep. Sleep analysis began on Day 7 of their second menstrual period following enrollment, during which participants wore an actigraphy wristwatch and completed twice-daily sleep diaries for 8 days and collected serial cortisol via dried urine samples for 4 days (Days 3-6 of sleep analysis). Created in BioRender. Jensen, K. (2025) https://BioRender.com/t88t094.

After establishing eligibility on a parent-reported screening form, eligible adolescents underwent an initial enrollment, which included an abbreviated *Structured Clinical Interview for DSM-V (SCID)* to screen for psychotic symptoms, bipolar disorder, and current suicidal ideation. During enrollment, participants and their parents were instructed on the daily and weekly assessment protocols and provided with filter paper strips for dried urine (cortisol and melatonin) collections. Starting seven days after menses onset, participants completed a one-week sleep and circadian rhythm assessment involving self-reported sleep behaviors, 24-h objective sleep monitoring using wrist actigraphy, and serial cortisol and melatonin (aMT6s) collections for four days to capture circadian rhythms during the mid-to-late follicular phase. The 8-day assessment period began 7 days after menses onset for all participants (Figure 1).

#### Study approval.

The study was conducted in accordance with the Declaration of Helsinki and was approved by the UNC Chapel Hill Institutional Review Board. Parents and adolescents provided written consent and assent prior to participating. Participants received prorated compensation for participation.

### Measures

#### Screening

Caregivers of prospective participants completed a brief online screen, followed by a phone screen. Screening questions included age, demographics, medical and psychiatric history, medications, and questions pertaining to pubertal development.

#### Baseline (enrollment) assessments

Participants completed a series of questionnaires assessing mood, interpersonal stress exposure, and sleep patterns at enrollment.

##### Pubertal staging.

Participants self-reported menarche status, breast development, pubic hair growth, height or growth spurt, and skin changes via the PDS using a 4-point scale, ranging from 1 (“No Development/No Menses”) to 4 (“Completed/Menses”). Self-assessment of Tanner Staging was completed using line drawings. A gonadal score was calculated as the sum of the height, breast development, and menarche items, according to (Shirtcliff et al., 2009). A category score was computed as the total sum of the scale values above, which, using cutoffs as described in (Carskadon & Acebo, 1993), can be used as a measure of pubertal progress (e.g., prepuberty, early-/mid-/late-puberty, and postpuberty).

##### Recent life events.

The *Coddington Life Events Record* (CLER) (Coddington, 1972) is a 26-item inventory of stressful life events adolescents may experience (e.g., parents’ divorce, gaining a sibling, a change in parents’ financial status, a major move, the death of a loved one). Participants selected items that they had experienced in the past six months. A sum score was calculated based on the total number of items selected. Higher scores indicate a greater number of stressful life events in the six months before enrollment.

##### Sleep characteristics.

Participants completed the 10-item *Sleep/Wake Problems Behavior Scale* (Wolfson & Carskadon, 1998) to assess sleep-related functional impairment (e.g., sleeping past noon) in the previous two weeks (scale: 1 (“never”) to 5 (“every day/night”), range: 10–50).

##### Chronotype.

The 10-item *Superscience Morningness/Eveningness Scale* (Carskadon et al., 1993) assessed chronotype (i.e., morningness vs. eveningness). Individual items were summed to obtain a total scale score ranging from 10 to 42, with higher scores indicating greater morningness and lower scores indicating greater eveningness.

#### Weekly self-reported assessments

Participants completed six weekly assessments of mood, sleep, and stress beginning on Day 1 of the first menstrual cycle following enrollment.

##### Mood report.

The *Center for Epidemiologic Studies Depression Scale for Children* (CES-DC) (Roberts et al., 1990) is a well-validated 20-item self-report assessing depressive symptoms in adolescents (scale: 0 (“Not At All”) to 3 (“A Lot”), range: 0 to 60). Higher scores indicate greater depressive symptoms, and a score of 15 indicates clinically significant depressive symptoms and likely diagnosis of MDD (Fendrich et al., 1990) – though it is important to note that the CESD-C is a screening tool and not validated as a diagnostic measure.

##### Sleep report.

The *PROMIS Sleep Disturbance Short Form 4a* and *Sleep-Related Impairment Short Form 4a* (Bevans et al., 2019; Forrest et al., 2018) assessed self-reported perceptions of sleep disturbance and daytime sleepiness, respectively, (scale: 1 (“not at all”) to 5 (“very much”), range: 4 to 20).

##### Perceived stress.

The *Perceived Stress Scale* (PSS) (Cohen et al., 1983) is a 10-item instrument assessing stress perception (scale: 0 (“never”) to 4 (“very often”), range: 0 to 40). The PSS consists of two subscales, one assessing *perceived helplessness* (negatively worded items) and the other assessing *perceived self-efficacy* (positively worded items) (Harris et al., 2023).

#### Daily self-reported assessments

Participants completed twice-daily sleep diaries (sleep–wake diary), where they self-reported bedtime, waketime, and variables that could impact sleep, such as caffeine use, exercise, social media use, stress, and daytime naps (Manber et al., 1996).

### Actigraphic sleep assessment

Following completion of the six-week main protocol (during which the above assessments were collected), participants wore an actigraphy wristwatch (Philips Respironics Actiwatch Spectrum Plus) for eight consecutive days to provide measures of sleep/wake indices, physical activity, and light exposure. The eight-day sampling period began one week following the start of a confirmed menstrual period, and allowed for inclusion of at least one full weekend in the SRI calculation. Participants were instructed to press the event marker button on the Actiwatch device upon waking each morning and just before falling asleep each night. Participants only removed the watch for water-related activities (e.g., bathing).

#### Actigraphy analysis and sleep characteristics.

Spectrograms were generated from the actigraphy data, which detailed activity, light levels, event markers, off-wrist periods, and sleep periods for each day (Philips Respironics, 2014). Rest intervals were confirmed by study personnel using a well-established procedure based on (a) self-reported bedtime/waketime, (b) activity drop/rise, (c) Actiwatch event markers, and (d) ambient light data (Dean et al., 2016). Individual 30-second epochs coded as either wake (1) or sleep (0) were generated using the Oakley algorithm (Oakley, 1997). Epochs outside of the main rest intervals that could not be determined automatically (referred to as excluded periods) were manually recoded as either wake or sleep, using the following procedure: (1) if the duration of the excluded period was less than 15 min, it was recoded as wake; (2) if the duration of the excluded period was greater than 15 min, the available self-report data regarding naps was used. When there was no evidence of naps, all excluded periods were recoded as wake. When a nap was reported, epochs were recoded as sleep beginning at the reported start time of the nap and continuing for the maximum reported duration of the nap (nap durations were reported as ranges [e.g., 15–30 mins]). Excluded epochs falling outside of the reported nap time were recoded as wake.

Cleaned sleep/wake epochs from the actigraphy files were used to calculate the SRI in Python using previously described methods (Lunsford-Avery et al., 2018). Other sleep markers were extrapolated from these epochs as well, including total sleep time (difference between waketime and bedtime, waketime-bedtime), sleep efficiency (percentage of time spent asleep while in bed), wake/bedtime variability (standard deviation of wake/bedtimes), wakefulness after sleep onset (WASO) (time spent awake between the bedtime and morning waketime), and sleep midpoint (midpoint between bedtime and waketime).

### Circadian rhythm measurement

#### Cortisol and 6-sulphatoxymelatonin.

On Days 3–6 of the sleep study (Days 9–12 of Cycle 2), participants provided four urine collections daily on filter paper: immediately upon waking (T1), 30 min after waking (T2), before dinner (T3), and before bed (T4). Participants were instructed to saturate the strip, record the exact date and time of collection, let the sample dry completely, and store samples in their home freezers until study personnel picked them up. Samples were then stored in the lab and kept frozen at -80°C until they were sent to ZRT Laboratory for analysis (ZRT Laboratory, Oregon, USA). Liquid chromatography-tandem mass spectrometry (LC-MS/MS) ensured the most sensitive and accurate quantification of urinary free cortisol and aMT6s. Average inter- and intra-assay precision for cortisol was 17.72% and 16.29%, respectively, and the reportable range was 0.7 to 275ng/mL. Average inter- and intra-assay precision for aMT6s was 8.83% and 6.26%, respectively, and the reportable range was 0.5 to 1588 ng/mL. Creatinine levels were used to correct values for hydration status.

### Analysis plan

#### Data coding and preparation

Sixty-six adolescents (ages 11–14, *M* = 12.8, SD = 0.96) provided data for the present analyses. Fifty-nine participants had acceptable sleep actigraphy (minimum six days total with at least one weekend day), and 62 participants provided cortisol/aMT6s samples for at least one day. Of the 62 participants with circadian rhythm data, 79% (*N* = 49) of participants collected the first sample within 30 min of verified waketime and the second sample within 45 min of the first to calculate the cortisol awakening response. Seven participants did not have actigraphy or self-reported waketimes to verify accurate collection times, and two participants had samples collected within 15 min of the verified waketime without a second sample within 45 min. Across all participants, 349 days of actigraphy data were collected with an average difference between actigraphy-measured and self-reported bedtimes and waketimes of 36.14 min (SD = 45.18) and 26.42 min (SD = 36.87), respectively. For CESD, missing values on individual questionnaires were imputed using mean imputation. Measure completion was 83% (327/396) across all study participants. Average cortisol and aMT6s levels at each time point are shown in Supp. Figure 1. Cronbach’s alpha was calculated as a measure of internal consistency reliability for relevant measures.

#### Diurnal cortisol/aMT6s

The collection time for each sample at T1 was compared to the validated waketime for the given day. Samples collected more than 30 min after the validated waketime were excluded from analysis. We calculated the diurnal slope (regression of values across the day onto hours since awakening, without T2) and the awakening response (CAR: difference between T2 and T1, T2-T1) (Ross et al., 2014) for cortisol. Samples were excluded from the CAR analysis if the time between T1 and T2 was greater than 45 min. For aMT6s, the level at T1 represented an estimate of overnight levels, as has been demonstrated previously (Arendt, 2006).

#### Statistics

A linear mixed effects model (PROC MIXED, SAS OnDemand for Academics) was used to examine relationships between depressive symptoms and SRI. Covariates included sleep duration, social jetlag, and age at menarche. Intercept was included as a random effect. A second linear mixed effects model assessed SRI-chronotype interactions using the same fixed effects as the direct model plus additional fixed effects of chronotype, self-reported stress, and the SRI*chronotype term. Restricted maximum likelihood estimation was used to estimate variance components. No additional imputations were made for missing data, as mixed models appropriately handle dependent variable data missing at random (Powney et al., 2014). Linear regression models were used to examine effects of cortisol (diurnal slope and CAR) and morning a6MTS levels on SRI.

All variables were treated as continuous for the primary analyses. For all models, assumptions of linearity, normality, and homoscedasticity were checked. The significance level was set at *α* =.05. False discovery rate was used to correct p values for multiple comparisons. Pearson correlations between sleep regularity with other sleep characteristics, including bedtime variability, total sleep time, sleep efficiency, sleep midpoint, WASO, and self-reported sleep impairment were conducted as exploratory analyses.

##### Power analysis.

With 66 participants, and *α* =.05, G*power (version 3.1) confirmed that we had 90% power to detect medium main effects (f^2^ = 0.15) of sleep regularity on affective symptoms and diurnal rhythms (cortisol, aMT6s), and 80% power to detect medium interaction effects of SRI with chronotype and on affective symptoms.

## Results

### Demographics and participant characteristics

Demographic and participant characteristics are presented in Table 1. Participants were predominantly White (69%), not Hispanic/Latina (90%), identified as female gender (90%), and were on average 8.6 months post-menarche. Average participant BMI percentile was 59.09 (*SD* = 25.81). Data were collected between March 2020 and December 2023, with an average of 23 months since the beginning of the COVID-19 pandemic at enrollment.

**Table 1.** Demographics

Participant characteristics	
Race (%)	
* White/Caucasian*	68.7
* Black or African American*	10.4
* Asian*	6
* Multiracial*	10.4
* Not specified*	3
Ethnicity (%)	
* Not Hispanic/Latina*	89.6
* Hispanic and/or Latina*	7.5
Gender (%)	
* Female*	92.5
* Nonbinary, gender fluid, cassgender*	4.5
Identify as LGBTQ+ (%)	
* Yes*	16.4
* No*	76.1
* Prefer not to respond*	4.5
Self-reported cycle regularity (%)	
* Regular*	36.2
* Neither irregular nor regular*	43.1
* Irregular*	27.6
* Unable to assess*	6.9
COVID-19 exposure (%)	
* No COVID-19 exposure*	73.1
* Positive COVID-19 test*	4.5
* Possible COVID-19 exposure*	19.4
	*Mean ± Std. Dev*
Time since COVID-19 onset (months)	23.22 ± 8.47
Age (years)	12.80 ± 0.96
BMI (percentile)	59.09 ± 25.81
Gynecological age (months)	8.56 ± 4.61
Age at menarche (years)	11.92 ± 0.92
Pubertal Development Scale	3.33 ± 0.44
Social media use (mins/day)	113.28 ± 73.20
Chronotype preference	27.52 ± 4.63

### Measure reliability

Reliability analysis demonstrated acceptable to good reliability across all measures, including CESD-C (*α* = .87), SuperScience Morningness/Eveningness (*α* = .78), PROMIS Sleep Disturbance (*α* = .82), PROMIS Sleep Impairment (*α* = .90), and PSS (*α* = .80).

### SRI and depressive symptoms

Summary statistics for mood measures are depicted in Table 2. In our direct model, reduced sleep regularity (SRI) was a significant predictor of weekly CES-DC depressive symptoms (*F* [1,276] = 9.74, *p* = < .002). No covariates in the direct model were significant predictors of depressive symptoms (*p* > .05).

**Table 2.** Descriptive statistics for assessments

Measure	Mean	Std. dev.
CESD-C		
PSS (NW)	7.63	4.29
PSS (PW)	6.63	2.78
Life events (CLER)	5.61	2.84
Anxiety (PROMIS)	14.55	5.46

CESD-C = Center for Epidemiologic Studies Depression Scale – Child Version; PSS = perceived stress scale, negative worded (NW = distress subscale), (PW = coping subscale); Life events = number of stressful life events on the Coddington Life Events Record (CLER); PROMIS =Patient-Reported Outcomes Measurement Information System.

### Chronotype as a moderator of SRI-affect

**Figure 2.**
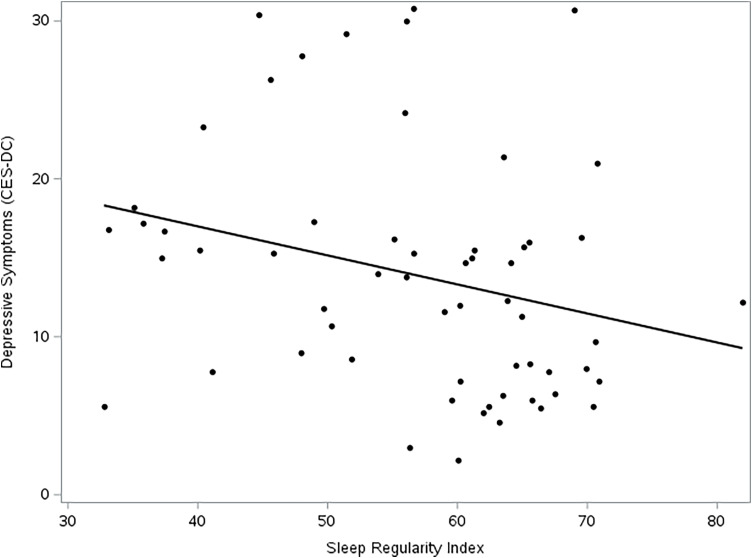
Depression vs. Sleep Regularity Index. Sleep regularity index reflects the probability of an individual being in the same wake/sleep state at any two timepoints 24 hours apart. Higher sleep regularity was associated with lower depressive symptoms.

The inclusion of stress, chronotype, and the stress × chronotype interaction increased model fit (AIC decreased from 2043.8 to 2001.2 compared to direct model). In terms of individual predictors, SRI (*F* [1,273] = 18.65, *p* = < .0001), chronotype (*F* [1,273] = 21.13, *p* = < .0001), and the interaction between SRI and chronotype (*F* [1,273] = 16.54, *p* = < .0001) were all significant predictors of CESD scores in the interaction model (Figure 2). Main effects of sleep duration (*F* [1,273] = 6.25, *p* = .01) and life stress (*F* [1,273] = 22.82, *p* = < .0001) were also observed.

### Sleep regularity and diurnal cortisol/aMT6s

Linear regression models testing the effects of the diurnal cortisol curve, cortisol awakening response, and aMT6s at timepoint one demonstrated no significant effects of endocrine circadian function on SRI (*p* > .05).

### Sleep regularity versus other sleep characteristics

See Table 3. Weak correlations were observed between SRI and TST (*r* = 0.15, *p* = .47), sleep efficiency (*r* = .02, *p* = .90), and WASO (*r* = .05, *p* = .87). Weak negative correlations were observed between SRI and sleep midpoint (*r* = −.26, *p* = .17), SRI and subjective sleep impairment (*r* = −.25, *p* = .18), and SRI and social jetlag (*r* = −.31, *p* = .10) (the absolute value of the average difference between weekday and weekend sleep midpoint (Sun et al., 2025)). A moderate negative correlation was observed between SRI and bedtime variability (*r* = −.52, *p* = .001).

**Table 3.** Correlation matrix of sleep measures

		SRI	Chronotype	Sleep dur.	Sleep eff.	WASO	Midpoint	Bedtime var.	Sub. sleep impair.	Social jetlag
*mean ± std.dev*										
**SRI**	r	1.00								
56.83 ± 11.29	p									
**Chronotype**	r	0.17	1.00							
27.52 ± 4.63	p	0.19								
**Sleep dur.**	r	0.15	0.26	1.00						
473.75 ± 39.28	p	0.26	0.05							
**Sleep eff.**	r	0.02	0.05	−0.30	1.00					
89.95 ± 3.52	p	0.88	0.72	0.02						
**WASO**	r	0.05	0.09	0.51	−0.86	1.00				
34.14 ± 15.26	p	0.70	0.49	< .0001	< .0001					
**Midpoint**	r	−0.26	−0.04	−0.16	0.21	−0.22	1.00			
0.12 ± .04	p	0.05	0.79	0.23	0.11	0.10				
**Bedtime var.**	r	−0.52	−0.30	−0.11	−0.08	−0.04	0.19	1.00		
.03 ± .02	p	< .0001	0.02	0.42	0.54	0.79	0.15			
**Sub. sleep impair.**	r	−0.25	−0.39	−0.08	−0.07	−0.01	−0.02	0.41	1.00	
51.65 ± 7.00	p	0.06	0.00	0.53	0.61	0.96	0.86	0.00		
**Social jetlag**	r	−0.31	−0.03	−0.19	0.12	−0.15	0.07	0.25	0.19	1.00
.03 ± .03	p	0.02	0.82	0.16	0.35	0.27	0.61	0.06	0.15	

*Note*. *r* = Pearson correlation coefficient; SRI = sleep regularity index; Chronotype = Chronotype preference (Superscience morningness/eveningness); Sleep Dur = sleep duration; Sleep Eff = sleep efficiency, total time asleep/total time in bed; WASO = wake after sleep onset; Bedtime Var = standard deviation in actigraphic bedtimes; Sub. Sleep Impair = subjective sleep impairment, sleep diary; Social Jetlag = absolute value of the difference between average weekend sleep midpoints and average weekday sleep midpoints.

## Discussion

### Summary of present findings

The current study examined sleep regularity in relation to mood and circadian rhythms in a cohort of early post-menarchal female adolescents. In line with previous findings, sleep regularity was shown to be a predictor of depressive symptoms. Unlike prior findings, however, greater sleep duration predicted greater depressive symptoms across the study period in our interaction model.

### Sleep regularity/duration and depressive symptoms

This study contributes to a growing body of research showing that sleep regularity plays a key role in adolescents’ emotional health. That this effect is present in early post-menarchal girls highlights the therapeutic potential of sleep regularity both to target acute mood dysregulation in the context of immature endocrine function around menarche, as well as to establish healthy trajectories for affective function to adolescence and adulthood. Consistent with other work (Phillips et al., 2017), sleep regularity was only weakly correlated with sleep duration, supporting the idea that sleep regularity and sleep duration should be considered as independent factors contributing to mood.

The longer sleep duration that was associated with higher depressive symptoms in our interaction model was likely a result of the eight days of sleep data being collected after the six-week mood assessment period, meaning we could not directly evaluate how changes in sleep duration related to mood at the time of assessment (i.e., sleep duration may have been different during the six-week mood assessment period). Additionally, adverse mood effects have been reported following experimental sleep deprivation (Johri et al., 2025), which utilizes more severe restriction than participants experienced in this study (the average sleep duration in this study was nearly 8 h).

### Sleep regularity, chronotype, and depressive symptoms

**Figure 3.**
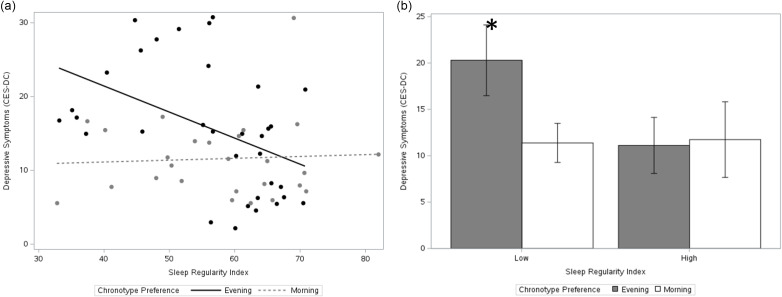
Interaction between Sleep Regularity and Chronotype in Predicting Depression. A. Interaction between sleep regularity index and chronotype in predicting depression scores. For visualization purposes, chronotype preference was categorized as “evening” or “morning” based on a median split of the continuous chronotype scores that were used in the primary analysis. B. Depression vs Sleep Regularity Index (categorical), grouped by chronotype *P<.005. For visualization purposes, categorical values for chronotype preference (morning/evening) and sleep regularity index (high/low) were obtained using a median split of their respective continuous variables (primary analysis used all variables in continuous form).

Regarding chronotype, eveningness preference was shown to be a risk factor for more severe depressive symptoms, consistent with prior studies (Murray et al., 2019). Additionally, an interaction between sleep regularity and chronotype were observed, such that eveningness and reduced sleep regularity together predicted higher depression. Dividing participants into high and low sleep regularity and chronotype at their respective medians suggests that depression scores were worse predominately for participants in the bottom 50th percentile for both sleep regularity and chronotype (Figure 3), meaning that female adolescents with an eveningness chronotype and sleep irregularity may stand to gain the most from a hypothetical treatment targeting SRI (though these findings should be interpreted with caution given the limitations of dichotomizing SRI in this manner). Given moderate inverse correlations between SRI and bedtime variability, increasing consistency of bedtimes may be one approach to improving sleep irregularity in “night owls.” Previous research has also suggested that earlier bedtimes in adolescents with delayed circadian phase may help normalize sleep function (Crowley et al., 2023), though our data demonstrated only weak correlations between SRI and sleep midpoint. A more individualized approach to parsing the effects of chronotype and behaviors that interfere with sleep timing might involve consideration of sleep phase angle – the time between the onset of evening melatonin secretion, which varies according chronotype (Taillard et al., 2021), and bedtime.

### Sleep regularity and circadian function

No significant association was observed for sleep regularity and steepness of diurnal cortisol slopes, cortisol awakening response, or overnight melatonin levels. Changes in cortisol dynamics (reduced CAR, flattened diurnal slopes) are thought to represent nonspecific stress-related dysregulation of endogenous circadian pacemakers (Adam et al., 2017) and have been linked to a host of mental and physical health conditions, including depression (Doane et al., 2013). Stress-induced alterations in HPA-axis function can disrupt circadian systems (Buckley & Schatzberg, 2005), contributing to mood dysregulation – further research is needed to clarify how sleep regularity relates by stress and stress-induced alterations in circadian rhythms.

### Sleep regularity and other sleep characteristics

Sleep regularity demonstrated a moderate inverse correlation with bedtime variability (about 27% of the variance), i.e., the consistency in participants’ bedtimes, and weak correlations were observed between SRI and sleep midpoint, sleep efficiency, and social jetlag, suggesting that other factors (behavioral, environmental, endogenous, etc.) contribute to sleep regularity, or that SRI reflects the combined effects of multiple influences.

### Strengths and limitations

The present study expands on prior work demonstrating relationships between sleep regularity, circadian timing, and mood (Lunsford-Avery et al., 2022; Murray et al., 2019; Walsh et al., 2025). Study strengths include the use of actigraphy as an objective measure of sleep, frequent assessment of mood and self-reported sleep characteristics, and the sampling of cortisol and melatonin (a6MTS) as a measure of circadian function. Despite the strengths discussed above, there are several limitations to address. Limitations include the relatively short study period without simultaneous ovarian steroid collection to confirm menstrual cycle phase, and a hormone sampling protocol with (a) a relatively small number of daily collections and (b) variability in collection times, due to home/participant-driven collection in adolescent participants. While all participants had a confirmed menstrual period one week prior to starting the study, reproductive hormone levels may have varied based on whether the cycle during which data was collected was ovulatory or anovulatory, which was not assessed in this study. In general, counting methods based on last menstrual period can be adequate for menstrual staging in regularly-cycling adults (Gloe et al., 2023), but are less useful in early post-menarchal samples given the high rates of anovulatory cycles; ovulation and/or hormone testing should be used when possible in this population. Recruitment was restricted to female adolescents to examine biobehavioral factors that may contribute to the increased prevalence of affective illness in females starting at puberty, which limited the generalizability of the results. Sex differences in sleep regularity, circadian function, and depressive symptoms will need to be examined in future studies. Additionally, though we included proximal measures of circadian rhythms in this study (cortisol, sleep midpoint, chronotype), direct assessment of the circadian clock, which requires serial melatonin assessment to establish dim-light melatonin onset, was not performed. Despite not employing the gold standard techniques for sleep assessment (polysomnography) or hormone sampling (venipuncture in a lab setting, allowing for greater control over sampling times and the ability to define hormone dynamics more precisely), we were nonetheless able to obtain high-quality sleep and endocrine data with a nonburdensome alternative with superior feasibility and compliance in adolescent participants. We intend to refine and expand this protocol for use in future experimental research.

### Future directions

Future research considerations include the use of experimental designs to elucidate causality, as well as testing potential treatments targeting sleep regularity. For instance, experimental paradigms for social stress (e.g., the Trier Social Stress Test), sleep deprivation, and circadian phase manipulation can be employed alone or in combination to assess effects on the HPA axis, sleep regularity, and affect. Further, combining ovarian steroid levels, circadian function, and sleep actigraphy across an adolescent menstrual cycle would offer important insight on the biobehavioral indices of depression risk in females. Samples that include a broader range of pubertal stages, as well as male participants, can increase the generalizability of findings. Denser hormone sampling protocols and the inclusion of melatonin would allow for more detailed assessment of circadian functioning, including cortisol awakening responses, melatonin curves, and phase angle, which can subsequently be examined in relation to sleep regularity. Longitudinal assessments can provide insight into the kinetics of mood, sleep regularity, and HPA-axis function, and may help us identify early stages of depressive illness that are amenable to sleep-based interventions.

From a clinical standpoint, the emergence of sleep regularity as a modifiable risk factor for affective function offers new possibilities for predicting and treating adolescent mental illness. Smartphone-based sleep tracking technologies may allow practitioners to roughly estimate sleep regularity in an office setting, providing insight into behavioral issues that adolescent patients may not be able to articulate precisely. Sleep regularity data may also help predict the onset of mood episodes and can be considered in addition to standard queries about sleep duration and subjective sleep impairment. From an intervention perspective, no treatments that target sleep regularity currently exist. However, interventions that address circadian timing, such as the Transdiagnostic Intervention for Sleep and Circadian Intervention, have been shown to have beneficial effects on mental health in adolescents with delayed circadian phase (Asarnow et al., 2023); given the purported relationship between circadian timing and sleep regularity, this and other chronotherapeutic interventions (such as bright light therapy) may stand to improve sleep regularity as well.

### Conclusion

The present study extends prior findings demonstrating that sleep regularity is an important risk factor for depressive symptoms in early post-menarchal female adolescents. Findings suggest that sleep regularity interacts with chronotype to influence mood outcomes. No statistically significant relationships were observed between sleep regularity and endocrine circadian rhythms, and future research may consider more intensive hormone sampling approaches. Interventions that target sleep regularity during peripuberty may directly improve adolescent depression, and positively impact long-term mental health trajectories.

## Supporting information

10.1017/S0954579426101242.sm001 Sikes-Keilp et al. supplementary material Sikes-Keilp et al. supplementary material

## Data Availability

The data and code necessary to reproduce the analyses presented here are publicly accessible, as are the materials necessary to attempt to replicate the findings. Data, code, and materials for this research are available at the following URL: https://osf.io/nmu5t/?view_only=a139ceca23a943be8fd42255952bb7d6.

## Supplementary material

The supplementary material for this article can be found at https://doi.org/10.1017/S0954579426101242.

## Pre-registration

Analyses were not pre-registered.

## AI

No AI tools were used in the preparation of this manuscript.
