# Supplementary material for: Sleep regularity, circadian rhythms, and chronotype as mechanisms of risk for affective dysregulation in female adolescents
Source: Dev Psychopathol. 2026 Feb 25:1–11. Online ahead of print. doi: 10.1017/S0954579426101242 (PMC12957652; doi:10.1017/S0954579426101242)
Supplement: Sikes-Keilp et al. supplementary material [file S0954579426101242sup001.docx]

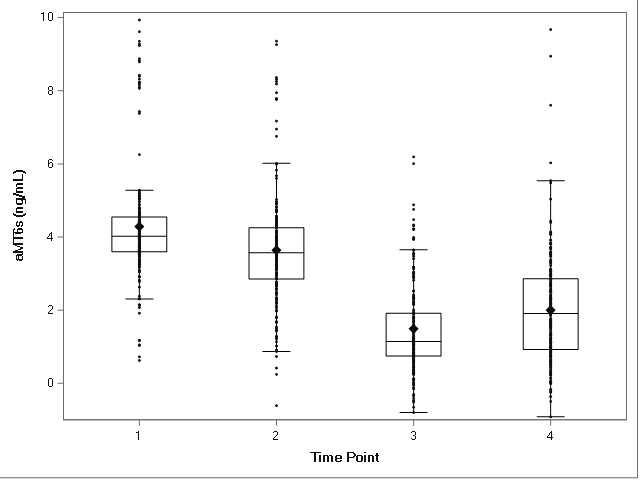

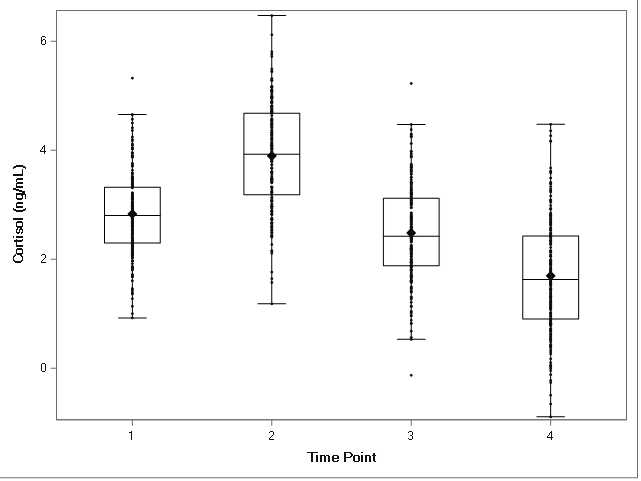


**Supp. Fig. 1. Average Cortisol/aMT6s Values by Time Point.** Horizontal lines across each box denote median values; diamonds denote mean values. Error bars reflect confidence intervals. For the daily time points, (1) was immediately upon waking, (2) was 30 minutes after waking, (3) was before dinner, and (4) was before bed.
